# Pegylated liposomal doxorubicin plus cyclophosphamide followed by docetaxel as neoadjuvant chemotherapy in locally advanced breast cancer (registration number: ChiCTR1900023052)

**DOI:** 10.1038/s41598-019-54387-5

**Published:** 2019-12-02

**Authors:** Ruoyang Li, Fuguo Tian, Yixin Qi, Li Ma, Tao Zhou, Yuntao Li, Tianli Hui, Lina Zhang, Shuo Wang, Zhenchuan Song

**Affiliations:** 1 Breast Center, Fourth Hospital of Hebei Medical University, Shijiazhuang, 050035 China; 2 Breast Center, Shanxi Province Tumor Hospital, Taiyuan, 030013 China

**Keywords:** Breast cancer, Breast cancer

## Abstract

Anthracyclines have a profound effect on breast cancer. However, at higher dosages, there are many toxic side effects associated with their use; these include bone marrow suppression, alopecia, gastrointestinal reactions and cardiotoxicity. Pegylated liposomal doxorubicin (PEG-LG) has been demonstrated to achieve equivalent efficacy to conventional doxorubicin, with significantly lower cardiotoxicity. We conducted an open-label, multicenter, single-armed clinical trial useing an NAC regimen based on four cycles of PEG-LD 40 mg/m^2^ plus cyclophosphamide (CPM) 600 mg/m^2^ on day 1 of a 21 day schedule, followed by four cycles of docetaxel (DTX) 85 mg/m^2^ on day 1 of a 21 day schedule. The primary endpoint analysed was the pathological complete response rate (pCR) in the breast, while treatment toxicities and safety were also assessed. The results showed that the breast pCR rate was 18.75% (95% CI 11.5–26.0%). Among the different molecular cancer types, the triple negative breast cancer patients had the highest pCR, at 43.75%. No significant decrease in left ventricular ejection fraction was observed. Our data tends to draw the conclusion that this regimen is a viable option for the neoadjuvant treatment of patients with LABC, especially in the triple-negative subtype and patients with heart abnormalities. We believe the efficacy and the safety of this regimen is likely to be the same based on published data from other studies but that this cannot be certain without a randomized trial.

## Introduction

Breast cancer is the most frequent malignant tumor type in women and is one of the leading causes of death in women worldwide^1^. In the current guidelines, most recommended chemotherapy treatments for breast cancer include doxorubicin; these include AC (doxorubicin, cyclophosphamide), FAC (fluorouracil, doxorubicin, cyclophosphamide) and FEC (fluorouracil, epirubicin, cyclophosphamide). Neoadjuvant Chemotherapy (NAC) with doxorubicin is now the standard treatment for locally advanced breast cancer (LABC). Due to the unfavorable growth kinetic characteristics of breast cancer metastasis, NAC possibly reduces micrometastases leading to an improvement in survival^2^. Furthermore, NAC is used to downstage tumors, facilitating breast conservation in patients who would otherwise undergo a mastectomy.

A meta-analysis of randomized clinical research showed that chemotherapy regimens that utilize anthracyclines to treat breast cancer are more effective than those that do not^3^. The NSABP-B27 study demonstrated that preoperative administration of docetaxel with anthracyclines increased the pathological complete response (pCR) rate from 13.7% to 26.1%^4^. Although anthracyclines have been shown to have a significant effect on breast cancer, increasing the dosage can lead to several toxic side effects such as bone marrow suppression, alopecia and gastrointestinal reactions. Furthermore, the risk of developing anthracycline-induced cardiotoxicity is related to several factors, but is primarily dependent on the cumulative dose of the agent^5^. Retrospective studies have shown that in addition to the accumulated dose, the risk of developing congestive heart failure (CHF) also correlates with the administration dose, whereby continuous doxorubicin infusion is less cardiotoxic compared with rapid infusion as this method prevents plasma concentration peaks^6^. Three observational studies demonstrated a significantly higher incidence of CHF with doxorubicin in women older than 65 years^7,8^.

Encapsulation of doxorubicin by pegylated liposomes (pegylated liposomal doxorubicin, PEG-LD) significantly modifies its pharmacokinetics and tissue distribution; this result in a comparable efficacy but a distinct toxicity profile compared to free doxorubicin^9^. Liposomal doxorubicin has an advanced occult liposome structure, a long cycle time and targeted enrichment in tumor tissue, which leads to significantly fewer side effects and a more potent anti-tumor activity^10^. The reason liposomal doxorubicin has a more favorable safety profile is due to two factors: less doxorubicin reaches cardiac cells, and additionally, the slower release of doxorubicin avoids peak plasma concentrations. The most probable cause is that liposomal doxorubicin has a longer half-life (50–80 h) than conventional doxorubicin (10 min)^11,12^.

With the development of precision medicine, it is necessary to develop new chemotherapy regimens with more convenient dosing schedules, better tolerability, and fewer side effects for patients. However, there are few studies investigating the use and dosage of PEG-LD in breast cancer treatment regimens. Our previous dose-escalating pilot study (NCT03017404) has confirmed that PEG-LG 40 mg/m^2^ is the maximum tolerated dose^13^.

To address this issue, we carried out this study (Clinical Trials registration number: **ChiCTR1900023052**), a phase II, open-label, multicenter, single-armed clinical trial aimed at evaluating PEG-LD plus cyclophosphamide (CPM), followed by DTX, as an NAC for breast cancer patients. This trial focusses on both efficacy and safety. We increase the sample size to determine whether it is safe to use in the neoadjuvant treatment and whether the efficacy is as good as we expected, in order to decide whether to proceed to a randomized controlled trial.

## Materials and Methods

### Eligibility criteria

The inclusion criteria for patients were that they must have untreated, histologically confirmed breast cancer. These candidates for NAC had tumors graded at stages II–III and had at least one measurable target lesion. The age range was ≥18 and ≤70 years old. Patients had a life expectancy of 12 months or more, as well as an Eastern Cooperative Oncology Group (ECOG) performance status of 0–2. All of the patients were required to have a left ventricular ejection fraction (LVEF) > 50%, as well as a biologic profile compatible with treatment (white blood cell count >4 × 10^9^/L, absolute neutrophil count >2 × 10^9^/L; platelet count >100 × 10^9^/L; creatinine between 44–133 µmol/L; bilirubin <1 time the institutional upper limit of normal (ULN); AST and ALT < 1.5 ULN; alkaline phosphatases <2.5 ULN).

The exclusion criteria were as follows: Patients who were pregnant or breast-feeding, had received any prior systemic or radiation therapy for breast cancer, had a history of allergic reactions to compounds of a similar chemical composition, had any other serious medical or psychiatric illness, any history of chronic liver disease, active uncontrolled infection, unstable cardiac pathology, or prior malignancies.

### Study design and approvals

Before the study progressed, all eligible patients signed a written informed consent form. The study was approved by the Fourth Hospital and the Ethics Committees of Hebei Medical University. All methods were performed in accordance with the approved guidelines and regulations. Furthermore, our study is registered at chictr.org.cn (ChiCTR1900023052, 9/5/2019). PEG-LD was obtained commercially (Shijiazhuang Pharmaceutical Group Ouyi Pharmaceutical Co., Ltd.).

### Study procedures

**Figure 1:**
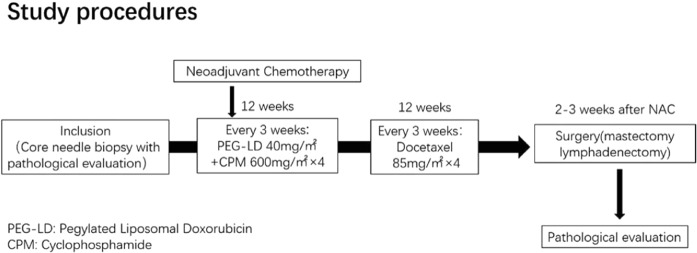
Scheme of this study.

This NAC regimen was based on four cycles of PEG-LD 40 mg/m^2^ plus CPM 600 mg/m^2^ on day 1 of a 21 day schedule, followed by four cycles of DTX 85 mg/m^2^ on day 1 of a 21 day schedule (Fig. 1). For patients with a HER2-amplified status, it was suggested they receive trastuzumab with DTX. Whether they use trastuzumab or not was according to the patients’ will (i.e. their financial status). Surgeons were asked to assess patient eligibility for surgery when completing NAC. Mammography, breast-ultrasound (US), and enhancement computed tomography (CT)/ magnetic resonance imaging (MRI) were performed at baseline to determine the diameter of the primary breast tumor. Baseline assessments also included a brain CT scan and bone scans for all patients. Laboratory evaluation (routine blood test, biochemistries, urinalysis, and tumor markers), an echocardiography and an electrocardiogram were also performed. Toxicity assessments were performed every 3 weeks during treatment and before surgery.

Tumor measurements were performed every 2 cycles according to Response Evaluation Criteria in Solid Tumors (RECIST1.1)^14^. Toxicities were graded using the Common Terminology Criteria for Adverse Events (CTCAE) v4.3. Treatment was discontinued in the case of: disease progression, unacceptable toxicity, the appearance of illnesses or clinical conditions which jeopardized continuation of therapy, or patient decision to withdraw from the study.

### Surgery and other adjuvant treatments

Surgery was performed between the second and third weeks after final NAC infusion. The options for surgical management included breast conserving surgery, mastectomy plus axillary dissection or sentinel lymph node biopsy; the choice was left to the surgeon according to best practice standards. Patients at high risk received adjuvant radiation therapy after surgery. Adjuvant anti-hormone therapy was given to patients with positive hormone receptors and adjuvant trastuzumab was given if the tumor was HER2-receptor positive.

### Pathological evaluation

Surgical specimens were evaluated by board-certified pathologists and assessed by immunohistochemistry, with a positivity cutoff of more than 1% for ER and PR. HER2 was considered positive if overexpression was scored at 3+ in immunohistochemistry or if the amplification ratio was >2 in fluorescent *in situ* hybridization. Breast pathologic complete response (pCR) was defined as no evidence of invasive cancer in the breast specimen. Axillary lymph node negative conversion rate was defined as confirmed lymph node metastasis before NAC, but absence of invasive cancer in the axillary nodes after.

### Statistical analysis

The primary end point of the study was the pCR rate in the breast. According to a recent study, it was assumed that there would be no interest if the pCR rate was ≤8% and of interest if the pCR rate >8%^15–19^. These assumptions were based on the pCR rate reported when treating with anthracyclines and taxanes for NAC in clinical trials before 2007^15–19^. Assuming a 10% drop-out rate, there should be 110 patients included in order to obtain 90 evaluable patients. The hypothesis test was resolved with a risk α = 0.05 and a power = 0.80, using a one-sample hypothesis test for proportion from π = 0.08 versus unilateral alternative, π > 0.08, based on the normal approximation. Secondary objectives were: complete response (CR), partial response (PR), objective response rate (ORR) before surgery, cardiac safety assessed by echocardiography (LVEF), electrocardiogram (ECG) and other toxicities. Rate values and 95% confidence intervals of each group were calculated respectively. Logistic regression analysis was used to test the difference of pCR rate among groups. Statistical description will be used as the main method.

## Results

**Figure 2:**
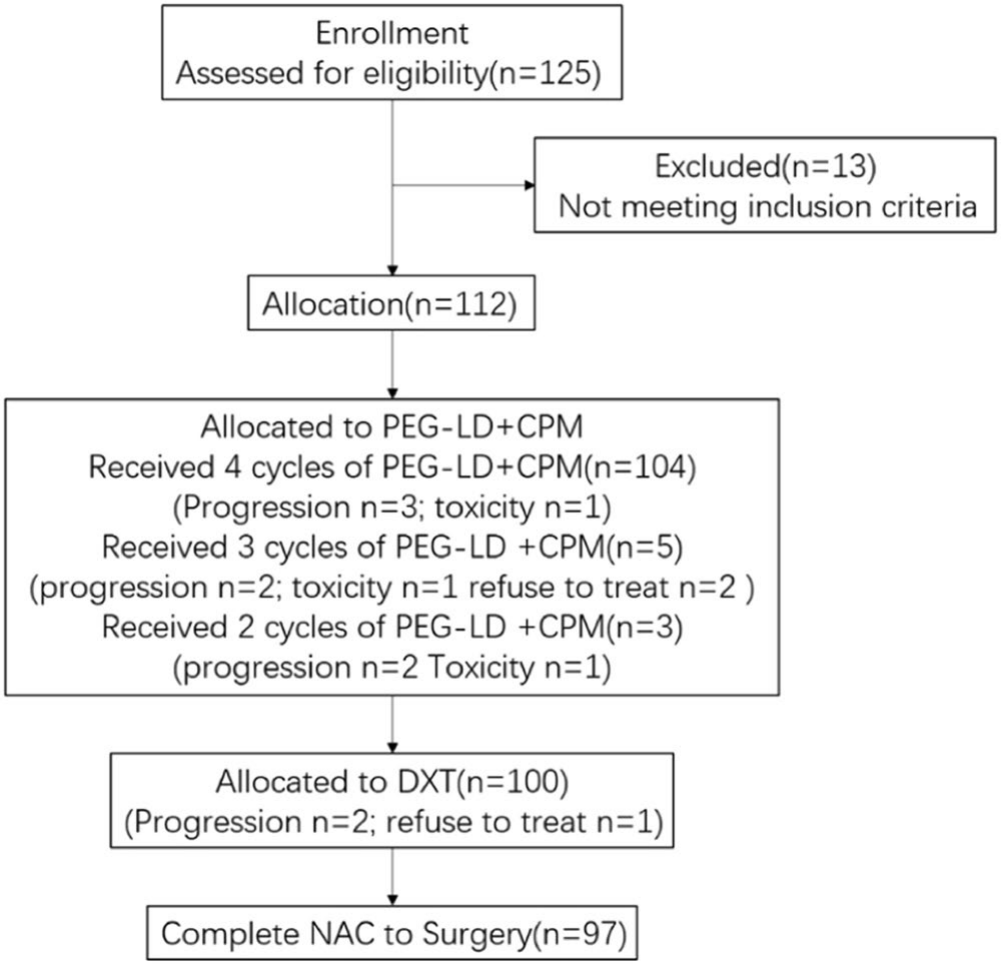
Flow diagram.

Between June 2017 and November 2018, 125 patients were enrolled from 2 breast cancer centers. 13 patients were excluded due to not meeting the criteria. 112 patients signed the informed consent form. 97 patients completed the NAC and underwent surgery. The drop-out rate was slightly higher than anticipated, however, the sample size was sufficient for statistical analysis (Fig. 2).

Patient and tumor characteristics are shown in Table 1. The median age was 47 years old (range 25–69). 104 patients (92.9%) completed the first four cycles of PEG-LD plus CPM. Meanwhile, 97 patients (86.6%) completed the NAC in full. 15 patients required dose reductions or withdrew from treatment (13.4% drop-out rate) (Fig. 2). All of the patients who completed the NAC underwent surgery (mastectomy and axillary lymphadenectomy) to examine their breast and axillary pathology status.

**Table 1:** Patient and disease characteristics.

	n	%
Patients	112	100
Age		
<40 years	25	22.32
40–45 years	23	20.54
46–50 years	22	19.64
51–55 years	16	14.29
56–60 years	13	11.61
>61 years	13	11.61
Menopausal status		
pre	75	66.96
post	37	33.04
Mean tumor size in mm(range)	38.7(19–120)	
Disease stages		
II	52	46.43
III	60	53.57
T clinical		
T2–3	100	89.29
T4	12	10.71
N clinical		
N0	14	12.50
N1	51	45.54
N2	13	11.61
N3	34	30.36
Molecular subtype		
Luminal A	9	8.04
Luminal B(her2-)	44	39.29
Luminal B (her2+)	20	17.86
Her2 (+)	23	20.54
Basal-like	16	14.29
Cardiotoxicity risk factors		
Age >65	1	0.89
Hypertension	16	14.29
Diabetes	11	9.82

The pCR rate in the breast was 18.75% (95% CI 11.5–26.0%). This result allows us to reject the null hypothesis (pCR ≤ 8%) with *p* <0.001 and prove the alternative hypothesis (pCR > 8%). 72 patients had been diagnosed with cancer metastasis in the axillary lymph nodes; 24 of these became negative post NAC, with an axillary lymph node negative conversion rate of 33.3% (95% CI 22.5–44.2%). Among the different molecular breast cancer subtypes, the triple-negative subtype (n = 16) had a much higher pCR, at 43.75%. In fact, pCR was observed in all breast cancer subtypes, with the exception of the luminal A subtype (Table 2). As for HER2+ positive patients, 8 of 20 patients with Luminal B (her2+) subtype received trastuzumab and 11 of 23 patients with Her2 (+) subtype received trastuzumab. Age, menopausal status, tumor size and T4 stage were analyzed as predictors of pCR by Chi-square test (statistical analysis by spss 23.0); no factor showed statistical significance (*p*  >0.05).

**Table 2:** pCR in different molecular subtypes.

Molecular subtypes	n	%	pCR(n)	%
Luminal A	9	8.04	0	0
Luminal B (her2−)	44	39.29	6	13.64
Luminal B (her2+)	20	17.86	4	25.00
Her2 (+):	23	20.54	4	17.39
Basal-like:	16	14.29	7	43.75
Total	112	100	21	18.75

pCR: pathological complete response.

Analyzing the secondary end points, 24 patients were defined as having a complete response (CR), 53 were considered to have a partial response (PR) and 26 were defined as stable (SD). ORR (77/112) was observed after PEG-LD–CPM plus DTX administration (summation of CR plus PR). Nine patients experienced disease progression (PD) during treatment with NAC (Table 3). All assessments were performed using imaging studies and in accordance to Response Evaluation Criteria in Solid Tumors (RECIST 1.1).

**Table 3:** Overall response in radiological.

Responses	Response after PEG-LD + CPM → DTX
Complete	24
Partial	53
Stable	26
Progression	9

PEG-LD: pegylated liposomal doxorubicin, CPM: cyclophosphamide, DTX: docetaxel.

**Figure 3:**
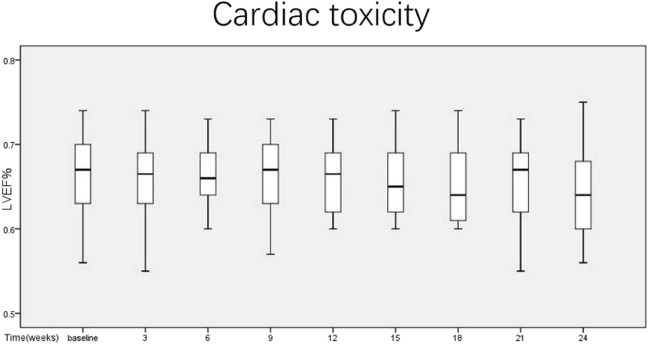
Mean LVEF over time from baseline, during the neoadjuvant treatment in ITT population.

No significant decreases in LVEF were seen across patients. The mean baseline LVEF was 67% (56%–74%), with mean LVEF post NAC at 65.9% (55%–73%) (Fig. 3). No Significant cardiotoxicity was observed in the study. No cases of left ventricular systolic dysfunction (LVSD) were reported, which was defined as an LVEF reduction to ≤50 percentage points, or a reduction in LVEF of more than 16 percentage points compared to the baseline value.

Table 4 displays the number of patients who suffered from treatment-related toxicities. Fifty-one (45.53%) patients developed hand and foot syndrome; this is in line with the findings of previous studies. Neutropenia (19.64%) and WBC decrease (25.89%) were observed less frequently than in previous studies. Other side effects included stomatitis (patients with constipation, 25% or diarrhea, 25.89%) and fatigue (74.11%).

**Table 4:** Most frequent toxicities.

Adverse event	All grades		Grade 3–4	
	n	%	n	%
Fatigue	83	74.11	0	0
Alopecia	72	64.29	0	0
Nausea/vomiting	91	81.25	0	0
Pain	44	39.29	0	0
Diarrhoea	29	25.89	0	0
Constipation	28	25	0	0
Anorexia	32	28.57	0	0
Fever	65	58.04	7	6.25%
Neutropenia	22	19.64	4	3.57%
WBC decrease	29	25.89	0	0
Hand-foot syndrome	51	45.53	16	14.28%
Infection	8	7.14	0	0
Cardiac event	5	4.46	0	0
Oral mucositis	44	39.28	0	0

## Discussion

As previously mentioned, doxorubicin use is limited by its potential to cause cardiotoxicity, particularly when cumulative doses are greater than 400 mg/m^2^. High cumulative doses can induce myofibrillar damage leading to a decrease in left ventricular ejection fraction, which can subsequently result in irreversible congestive heart failure^20^. Encapsulation of doxorubicin by pegylated liposomes has been shown to achieve equivalent efficacy to conventional doxorubicin, with significantly less cardiotoxicity^21^. There has been a growing interest in the use of PEG-LD with cyclophosphamide in patients who are considered too frail/unsuitable for conventional doxorubicin treatment^22^. A recent study found that PEG-LD, in combination with daily oral cyclophosphamide, is an effective and tolerable regimen for the treatment of metastatic breast cancer^21^. In the phase I study we conducted, it was demonstrated that PEG-LD 40 mg/m^2^ and CPM 600 mg/m^2^, followed by DTX 85 mg/m^2^ on day 1 of a 21-day schedule, is an efficacious and well-tolerated neoadjuvant regimen for patients with locally advanced breast cancer. In the phase II trial presented here, we used the same regimen of NAC to evaluate the pCR, assessing the efficacy and safety as in standard doxorubicin treatment.

Previous studies have demonstrated that the combination of anthracyclines and taxanes, either sequentially or concurrently, lead to a high rate of pCR (8–30%) in patients not selected by histological subtype^15–19^. In our study, the total breast pCR was 18.75% (95% CI 11.5–26.0%); this is similar to pCR levels found in previous research. One reason for a slightly lower pCR in our study may be due to the selection of patients with a higher proportion of advanced cancer (most are stage III). To our knowledge, at time of writing, no standardized definition for pCR exists. Some recent trials have used the pCR definition of ypT0/is^23^, whereas others included axillary node pCR (ypT0/is ypN0) to determine the effectiveness of the regimen^15^. The axillary lymph node negative conversion rate was 33.3% (95% CI 22.5–44.2%). Among the molecular subtypes of breast cancer in this study, the triple-negative subtype had a much higher pCR, at 43.75%; in comparison, the luminal A subtype had no evidence of pCR. This study was not strictly designed to assess the breast pCR of different subtypes; however, we believe this observation shows luminal A patients benefit significantly less than patients with other cancer subtypes, with the triple-negative patients benefitting the most.

Prior research has already demonstrated that PEG-LD is an effective first-line agent for metastatic breast cancer with recent studies focusing more on NAC^24^. One trial compared 43 women who received neoadjuvant chemotherapy containing PEG-LD to those who received regimens containing epirubicin; the results showed that PEG-LD treatment resulted in a similar clinical response rate (76.7% vs 75.6%)^25^. In a phase II trial, the efficacy of PEG-LD 35 mg/m^2^ plus CPM 600 mg/m^2^ every 3 weeks for three cycles was assessed in 30 patients with locally advanced breast cancer. The ORR was 73%^26^; In our study, 77 of 112 patients had achieved CR or PR with an ORR of 68.75%, slightly lower than the average.

The most serious adverse effect of anthracyclines is cardiotoxicity. To the best of our knowledge, there is no normalized assessment for anthracycline-induced cardiotoxicity. LVEF baseline evaluation, with repeated assessment periodically during the treatment, is the most widespread clinical practice for the estimation of toxicity to the heart. Our study showed no evidence of a significant decrease in median LVEF (67.05% before, 65.91% after, *p* > 0.05). There were also no relevant changes in ECG during chemotherapy cycles. Five patients had occasional ventricular tachycardia during the ECG, without any further symptoms. However, it’s worth noting that the administration of PEG-LD 50 mg/m^2^ plus trastuzumab every 4 weeks for six cycles in patients with HER2-positive metastatic breast cancer resulted in the development of grade 1 cardiotoxicity in three patients (10%), without any symptomatic CHF^27^.

Additional typical toxicities associated with PEG-LD are HFS, skin reactions, and mucositis^28^. PEG-LD 40 mg/m^2^ every 4 weeks appears to offer the most favorable toxicity profile^29^. In our previous study, PEG-LD 40 mg/m^2^ every 4 weeks was shown to be less toxic than other treatment regimens. Using this sequential regimen, Neutropenia was documented in 19.64% of our patients, with only 4 cases at grade 3–4 (3.75%). It is known that PEG-LD -induced dermal toxicity is linked to cutaneous accumulation, most likely due to the long half-life of the drug. Damage of the vasculature occurs, which subsequently results in extravasation of the cytostatic compound into the surrounding tissues. With the use of PEG-LD, HFS was seen in 51 (45.53%) patients in our study. 16 (14.28%) patients were Grade 3–4. This proportion is within the range of prior studies using PEG-LD 35 mg/m^2^ plus CPM^30,31^ or PEG-LD 45 mg/m^2^ ^32^ every 4 weeks; and is much lower than a study utilizing PEG-LD 50 mg/m^2^, C*PM* 600 mg/m^2^ plus trastuzumab every 4 weeks^33^. Of all the 51 patients who developed HFS, nearly half also suffered from stomatitis; which was improved with a treatment delay and subsequent dose reduction. Regarding other toxicities, neutropenia and WBC decrease were the most common G1–2 adverse effects that we identified in this study. Patients with these side -effects rapidly improved after treatment with antibiotics and recombinant human granulocytecolony stimulating factor (rhG-CSF). Stomatitis (constipation and diarrhea) was a frequent adverse event in this study (50%), although it never exceeded grade 1–2 in any patient. Fatigue was present in 83 patients (74.11%), which correlates with the findings of previous studies.

## Conclusion

In summary, we utilised a breast cancer treatment regimen that included PEG-LD 40 mg/m^2^ and cyclophosphamide 600 mg/m^2^ followed by docetaxel 85 mg/m^2^ on day 1 of a 21-day schedule. This achieved a pCR rate in the range of conventional anthracycline plus taxane-based regimens. Toxicity was lower than those using other analogues and the treatment was found to be acceptable for the majority of patients. Our data incline to conclude that this regimen can be utilized as an alternative option for the neoadjuvant treatment of patients with locally advanced breast cancer, especially in those patients with the triple-negative subtype and in those who cannot tolerate the routine anthracyclines. We believe the efficacy and the safety of this regimen is likely to be the same based on published data from other studies but that this cannot be certain without a randomized trial.
